# The Relation of Diseases

**Published:** 1881-09

**Authors:** P. W. Van Peyma


### The Relation of Diseases.*

By P. W. Van Peyma.

As nuclei for the development of the subject to which your attention is called, I shall make use of two quotations, both from earlier papers of mine. The subject of one is "Inflammation," that of the other "Malignancy in Tumors." The first was read January 9, 1877; from it we take the following: "It has been my object to give a rational explanation of the phenomena of inflammation. I have not attempted to determine

*Read before the Medical Club, July 20, 1881.

when, in the process which we have been considering, inflammation really and exactly begins, or when it as precisely ends. Believing the transition from one condition to another gradual, and the lines separating inflammation from simple hyperæmia on either side arbitrary, this would have been inconsistent. And in conclusion I will say that to my mind inflammation is not alone in the fact of its gradual transition into other conditions. My conviction is settled that as we progress in our knowledge of the general principles of disease, the application of this law of transition will be found to approach the universal."

The second, from a paper read two years later, in 1879, is to the same effect: "In the study of malignancy we are again reminded that in nature there are no sudden jumps from one extreme to another, but always a gradual transition. One kind of morbid action gradually merges into another."

From these quotations and the dates at which the papers containing them were read, it will be seen that this idea of transition is not simply a temporary or ephemeral hobby, but a fixed belief, which, I may add, time has but strengthened. For this reason and also because, although a theoretical subject, it treats, as I believe, of principles and has a direct and important practical bearing, I have considered it worthy of the attention which you may give it. It cannot be denied that the truth or falsity of the views brought forward must have a serious practical bearing. None the less is it true that the positions must stand or fall by what we shall in the future learn or unlearn touching many practical points, such as the causes and relations of diseases.

The purpose of this paper is to show that the classification of diseases and, in many instances, the conceptions of their nature are surely and entirely arbitrary matters, depending upon individual notions—the individual and collective personal equations. This thought will be developed to show that pathological processes show transitions into those of similar character, and that where we draw the lines of separation, in fact, that we draw the lines at all, indicates a wrong conception of the laws of nature in general and of pathology in particular. This subject, like all of its class, admits of two methods of examination.

First, then, we may commence with our knowledge of diseases as they present themselves in our time, and secondly, commencing at what we assume to have been the primitive condition, we may by reasoning attempt to fill in the intervening condition of things. If by these means we should fail to make the lines of reasoning meet, we shall still expect to approximate them with perhaps here and there a missing link. Has not the attention of every one here present been forcibly called to the difference between the descriptions in text-books of typical cases and the anything but typical cases we meet with in daily practice? Were text-books our only guide, we must come to the belief that each disease is a sharply-defined entity, abruptly separated from all others. And this is apparently the view adopted, perhaps without more reflection, by the profession generally. We should, according to this view, naturally expect, for example, erythematous and follicular pharyngitis, quinsy or tonsilitis, malignant or putrid sore throat, and diphtheria, all to be diseases entirely distinct and presenting each a morbid process peculiar and pathognomonic. Again, that hyperæmia and inflammation are to be looked upon as essentially distinct; that malignancy is something specific. In the sphere of the germ theory of disease each particular form of bacteria to be the source of its own characteristic pathology. If this be the correct view, leucorrhœa can have no connection with gonorrhœa; no more can dumb ague, remittent, intermittent, typho-malarial and pernicicus intermittent fevers have any relation to each other.

Is this a true picture of what we see at the bedside? I think not. That typical cases of different diseases bear but slight, if any, resemblance to each other, we of course do not dispute; but many, perhaps the majority, of cases are not typical, and it is in these that we think to find the steps of transition. That there is more contrast than comparison between diphtheria and erythematous pharyngitis, for example, is obvious; but when between these two extremes we place the other inflammations of the pharynx, we are enabled to follow the pathological variations step by step. Cases of fever with the mixed rash of measles and scarlatina, and with one or more of the usual concomitant symptoms of either, are equally suggestive.

To bring the subject within the scope of this paper, we must here limit our first line of argument to take up the second. This latter we open with the proposition that disease must of necessity depend upon one or both of two causes, or rather groups of causes; the one those of heredity, the other those of active circumstances, such as air, temperature, food, etc. This being admitted, the converse follows that a perfect man in a perfect environment must enjoy undisturbed and perfect health. Like the "one-hoss shay," he would perhaps live "an hundred years to a day," and then like that would go, nothing first, but all at once. Like a candle when burned to its socket, he would "go out" for the reason that the tissues are oxidized and blown away. Such a man might Adam have been in Paradise—a perfect man in perfect surroundings.

Now it is evident that, although the cell is originally perfect, circumstances may, during its growth, result in aberration from the normal, and this is what we call disease. Authors have apparently found great difficulty in defining health. The reason for this is found in the fact that the line must be drawn at the first moment of aberration, or else it becomes an entirely arbitrary matter. This point, although very important, does not appear ever to have received much attention. Of course it follows, as we well know, that no human being, since the ideal and mythical Adam, has ever enjoyed perfect health. We are all sick; we are all insane. How much discussion might have been avoided if this unavoidable truth had been aknowledged? Who can tell exactly where eccentricity ends and insanity begins? The answer is, every doctor, and it might be added, "every old woman," but let it also be added no two alike. Where does benignancy end and malignancy begin? Both the answer and the apparent criticism are the same. Examples could be multiplied indefinitely. Does not the thought suggest itself that classification is arbitrary and that transition is the explanation? Is it not evident that a gradual, almost if not quite insensible, transition exists between the various forms of disease? This we maintain and shall endeavor to prove. For this purpose we shall adopt the mode of reasoning employed in the demonstration of the non-existence of a so-called free will. Three factors require consideration: first, perfect man; second, natural man; and third, the circumstances in which he lives.

A human being is the sum of what he was when born plus the effects of influences which have acted upon him during his life-time. Every human being is the sum of a perfect man plus the effects of influences which have acted upon him and his ancestors.

We begin, therefore, with a perfect man, and shall endeavor to follow the changes to which he is subject as a result of external causes acting upon him. These are of course manifold. The food he eats may be improper in quantity for quality, the water he drinks either too cold, or hot, or impure in character, and so on. As one of the simplest in its consequences let us follow the results of an abnormally increased temperature of the atmosphere. The heart increases in rapidity, the skin becomes more active, in short, all the vital functions are hastened. The man is simply unhealthy in that he lives too fast; he will run down before the hundred years have run their course.

We now add another abnormal condition—the atmosphere contains an abnormal amount of vapor. The result is clear. The skin and the organs no longer act as safety valves; the temperature of the person increases. He has fever. These are still comparatively elementary conditions, and before following them further it will be well to add one or more additional illustrations.

Our man submits different portions of his body to unequal temperature, or gives them unequal opportunity for retaining or radiating the animal heat. He sits in a draft of cold air. The parts abnormally cooled contract, be this contraction physical or through the influence of the nervous system, the result is an approach to localized anemia. This, if continued, or becoming intense, results in appreciable diminution of nutrition. On the other hand the irritation of a foreign body results in increased localized vital activity, the assimilation and disassimilation no longer corresponding, and a local hyperplasia or tumor is formed. Usually, however, the irritation of a foreign body is more violent, the hyperæmia continues to increase, the serum of the blood is transuded, followed, perhaps, by albumen and blood, the proliferation becomes more and more rapid, the cells are hastily and imperfectly formed, they infiltrate the tissues interfering with the circulation, fatty degeneration takes place, the part becomes disorganized, breaks down, and we all call it inflammation. Are we not right in calling this a progressive, a transitional process?

At present we are much inclined to explain many morbid conditions, by referring them to disease germs, just as years ago everything was explained by recourse to the theory of reflex action. In both instances physicians, in their eagerness to profit by the latest researches in physiology and pathology, have undoubtedly gone too far towards one extreme, as they will surely in turn go to the other. Still, evidence is accumulating, tending to prove that bacteria and kindred forms of the lower organisms are in numerous instances active causes of disease. The varieties of bacteria (using the word in its broadest sense) and the character of their activities, chemical or vital, are as yet too little understood for us to enter deeply into the subject. But from what is known it seems probable that their action may be compared to that of other irritants. The collection of symptoms, which we designate as febrile, is almost invariably present in diseases attributed to their agency. If, as is probable, variations of the character of germs—that is of the cause—result in variations in the effect—that is in the disease—then we may rightly assume that if the cause is transitional, so will be the disease. Our knowledge of animal and vegetable life, both the higher and lower forms, leaves no doubt that such transition actually has taken place during the ages of the past. We infer, then, that such has been the case in this group of diseases. We further conclude that as animal and vegetable life tends constantly to differentiation from conditions of uniformity to those of variety, that the same is true with this group of diseases. At this stage of the world's history we would expect, as we in fact find, that many diseases have assumed a form quite characteristic and peculiar. But here and there we still find the links which connect them with other forms less developed and differentiated. That new forms of disease have in the past and will in the future continue to make their appearance, is in perfect accordance with this view of their origin and course. That the limited number of pathological processes, such as inflammation, degenerations, etc., into which all others may be analyzed, involve a universal law, I firmly believe. Were this otherwise, it would be in contradiction with all we know of the laws of nature of anthropology, biology, and all the kindred departments of knowledge.

This thought can perhaps be made clearer by imagining a sphere whose center is health and whose circumference is death, with lines radiating from the center to various points of the surface. These lines to be considered as lines of disease. The graver forms approaching the surface, while the milder scarcely leave the center of health. At the center the lines are closely approximated, while at the circumference they are widely divergent. So with disease: first closely resembling each other, afterward by a process of gradual differentiation they have become to a great degree dissimilar.

The arguments drawn from our daily clinical observation and those founded on the nature of things both point, then, in the same direction, towards establishing the truth of the proposition that all forms of disease are transitional.

For want of time this simple outline of the argument must suffice.

In conclusion we will add a few words touching the practical bearing of the subject.

The results of a general adoption of these views as the truth must incline in the first place to a broader and more scientific conception of health and disease. While we recognize the classification of disease in some such way as the present, as an advantage in description and for the purpose of calling attention to incidental complications, we shall bear in mind the facts of its arbitrary character.

As a result we see in the future that treatment is more and more based on general principles, generally applicable, and varied only as demanded by special circumstances. Specifics are less prized and sought for, and when found, are more generally applicable. Less stress is laid upon the name and inversely more upon the essential nature of the difficulty. As our conception of the nature of diseases improves, so will our treatment, and this being the ultimate object of all medical art and science, we can clearly see the practical importance of the views which we have endeavored to maintain.

